# Natural Polymers as Green Binders for High‐Loading Supercapacitor Electrodes

**DOI:** 10.1002/cssc.201902863

**Published:** 2020-01-21

**Authors:** Peter Ruschhaupt, Alberto Varzi, Stefano Passerini

**Affiliations:** ^1^ Karlsruhe Institute of Technology (KIT) P.O. Box 3640 76021 Karlsruhe Germany; ^2^ Helmholtz Institute Ulm (HIU) Helmholtzstrasse 11 89081 Ulm Germany

**Keywords:** binders, carbohydrates, flexible electrodes, high mass loading, supercapacitors

## Abstract

The state‐of‐the‐art aqueous binder for supercapacitors is carboxymethyl cellulose (CMC). However, it limits the mass loading of the coatings owing to shrinkage upon drying. In this work, natural polymers, that is, guar gum (GG), wheat starch (WS), and potato starch (PS), were studied as alternatives. The flexibility and adhesion of the resulting coatings and electrochemical performance was tested. The combination of 75:25 (*w*/*w*) ratio PS/GG showed a promising performance. Electrodes were characterized by SEM, thermal, adhesion, and bending tests. Their electrochemical properties were determined by cyclic voltammetry, electrochemical impedance spectroscopy, and cycling experiments. The PS/GG mixture conformed well to criteria for industrial production, enabling mass loadings higher than CMC (7.0 mg cm^−2^) while granting the same specific capacitance (26 F g^−1^) and power performance (20 F g^−1^ at 10 A g^−1^). Including the mass of the current collector, this represents a +45 % increase in specific energy at the electrode level.

## Graphical abstract

**Better binders**: Carboxymethyl cellulose (CMC) is the state‐of‐the‐art aqueous binder for carbon‐based supercapacitors, but it is inflexible, shrinks during drying, and cracks at high mass loadings. Mixtures of natural carbohydrates from potato starch and guar gum enable flexible yet high‐mass‐loading electrodes. Their electrochemical stability and performance are equivalent to CMC.

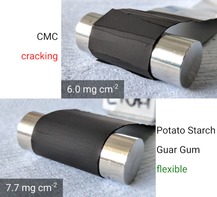

## Introduction

Electrochemical double‐layer capacitors (EDLCs) are high‐power energy‐storage devices offering long cycle life. Numerous unique applications already exist for EDLCs, and their use in the expanding ecosystem of renewable energy is growing steadily as more and more effort is put into fighting climate change. Their energy‐storage mechanism is based on the fast and reversible adsorption of ions from an electrolyte onto a porous high‐surface‐area material. Commonly, commercial EDLCs include activated carbon (AC) electrodes and organic electrolytes, which reach approximately 3 V operating voltage.^1^

Production of these devices involves coating large sheets of metal (Al) current collectors by using fast, low‐cost, roll‐to‐roll processes. Coatings are applied as slurries consisting of the active material, conductive additive(s) (carbon black, CB), and polymeric binder(s). This latter must fulfill several requirements,^1^, ^2^, ^3^, ^4^ such as:

1. gives uniform coatings on the Al current collector;
2. endures drying at high temperature in air without degrading;
3. provides mechanical stability (the resulting coatings have to survive mechanical stresses such as bending and rolling against steel rolls, pressing in a calender and, finally, tight winding in cylindrical cells);
4. grants an optimal microscopic structure of the electrodes, with well dispersed conductive additives to reduce resistance;
5. is electrochemically inert to not jeopardize the operation of the EDLC;
6. requires an environmentally friendly solvent (such as water) for slurry processing or, ideally, no solvent at all.

Despite some alternatives having been recently proposed, the state of the art in aqueous, non‐fluorinated binders for EDLC is carboxymethyl cellulose (CMC).^2^, ^5^, ^6^ Although this material conforms to the requirements outlined above, it has one major drawback, which is that the maximum thickness (or mass loading) possible for coatings is relatively low. The reason for this lies in the shrinking of the CMC coatings upon drying, which can result in cracking of the coating. Additionally, the low flexibility of the coating at high mass loadings causes cracking upon, for example, rolling after drying. Also, electrodes based on alternative water‐soluble polymers, such as poly(acrylic acid) (PAA) or sodium alginate, have been only reported with relatively low mass loadings (<5 mg cm^−2^),^7^ suggesting that such binders may suffer from the same issues as CMC. Achieving higher mass loadings is extremely important because it would allow higher active material/current collector mass ratios, consequently improving the specific energy of EDLCs.^1^ In this work, new binders based on water‐soluble natural polymers were comprehensively studied. The study allowed the identification of a promising new binder mixture fulfilling the above‐mentioned criteria and substantially outperforming CMC in terms of flexibility and maximum mass loadings of the resulting electrodes.

## Results and Discussion

### Coating tests

A few binder candidates [cold‐water‐soluble wheat starch (WS), potato starch (PS), and guar gum (GG)] were investigated for the preliminary coating tests. This served to check the basic suitability of the materials as possible binders. Starch is a natural polymer consisting of two types of polysaccharides, that is, amylose and amylopectin. The first is constituted by α(1–4) glycosidic‐bonded α‐d‐glucose units forming a linear chain. Amylopectin is similar, but with many additional side chains connected by α(1–6) glycosidic bonds. Their mass ratios, chain lengths, branching, and other factors depend on the botanical source. These polysaccharides are produced by plants in the form of so‐called “granules”, that is, particles of 2–100 μm in size. They also contain varying small amounts of protein and minerals.^8^ In the case of cold‐water‐soluble starch, these granules are shredded or predissolved to increase their solubility in water without need for elevated temperatures.^8^, ^9^ GG is also a polysaccharide produced from guar beans. It differs from starches in that the backbone is made from β(1–4)‐bonded d‐mannopyranose units carrying short side chains on every second unit. These side chains are single (1–6)‐bonded α‐d‐galactopyranose units. GG is known to strongly increase the viscosity of aqueous solutions even at low concentration, for which it is used in a wide range of applications from food additives to fracking.^10^

Each binder candidate was mixed with water, active material, and conductive additive to yield 5 wt % binder content with respect to all solids in the slurry. Three qualitative criteria were applied for the initial screening:

1. Processability of the slurry: the slurry should not show signs of sedimentation within the timescales needed for the coating process (1–3 h).
2. Uniformity of the coating: the slurries needed to produce homogeneous coatings without bubbles, pinholes, stripes, or similar defects; the coating quality could typically be modified by the addition or removal of water.
3. Solid content of the slurry: binders not enabling sufficiently high solid content (>25 wt %) but fulfilling the previous two points were excluded.

These criteria result from the needs of cell production. In fact, homogeneous coatings are indispensable to ensure tight wrapping of the electrode and separator rolls, which in turn ensures precise balancing and low performance variation between cells. High solid contents are desirable because any excess solvent needs to be evaporated before further processing. The more water is in the slurry, the longer (slower) the drying line has to be (run), which results in higher costs and reduced productivity, respectively.^1^ Settling of solids in the slurry can cause density gradients in the slurry container. This would cause the properties of the slurry to change during coating and therefore cause inhomogeneous coatings.^7^

The results of the binder screening are summarized in Table 1, including those of the bending test discussed in the next section. Additional photographs can be found in Figure S1 in the Supporting Information. It should be noted that none of the binders are found to be entirely suitable on their own. The typical high viscosity induced by GG reduces the maximum possible solid content for coatable slurries to only 20 wt % (Figure S1 A in the Supporting Information). WS‐based coatings are brittle and show poor adhesion at all mass loadings (Figure S1 B in the Supporting Information). Homogeneous WS and PS slurries typically have low solid contents and therefore result in low mass loadings even when applied with large doctor blade slit heights. Using higher solid contents is not possible because mixing does not result in homogeneous slurries (Figure S1 C in the Supporting Information).

**Table 1:** Screened binders and criteria of evaluation. Underlined entries mark the reason for exclusion.

Binder	Maximum solid content [wt %]	Coating defects	Adhesion	Mass loading passing bending test [mg cm^−2^]
GG	20	pinholes	good	>5
WS	25	stripes	insufficient	≤2
PS	25	stripes	good	>4
WS/GG (1:1, *w*/*w*)	28.6	none	insufficient	<6
PS/GG (3:1, *w*/*w*)	31.6	none	good	<7.5

However, mixtures of both starches (WS and PS) with GG allow up to 31.6 wt % solid contents, suggesting synergistic effects of the two binders. GG serves to enable slurries with higher solid contents, acting as an emulsifier and stabilizer, allowing for a much easier slurry mixing. In fact, the advantageous interaction of GG with starches is well known in the food industry, in which it is used, among other things, to reduce the starchy texture of soups and avoid syneresis (the expulsion of water from the polymer network).^11^, ^12^, ^13^ This interaction is also evidenced by the tendency of slurries to gel reversibly when left standing. However, the perturbation owing to stirring and pumping in industrial coating lines would easily disrupt the slurry gelation because simple stirring before coating was always sufficient to re‐homogenize the slurries. The two most promising mixing ratios, also with respect to the coating flexibility (see below), were 1:1 for WS/GG and 3:1 for PS/GG. They are denoted as WS50/GG50 and PS75/GG25.

### Bending tests

Besides the rheological properties of the slurry, which were here only qualitatively monitored, the most important criterion for the binder selection is the morphological and mechanical quality of the resulting coating. In the industrial environment, after drying, the coated current collector foils are, in fact, handled via transport rolls and finally rolled up for intermediate storage. This means that the coating must not crack during bending and must be able to endure without too much abrasion, which is particularly critical for high mass electrode loadings. Achieving high bending flexibility is the key here because thick coatings easily break at small curvature radii.^1^, ^14^

**Figure 1:**
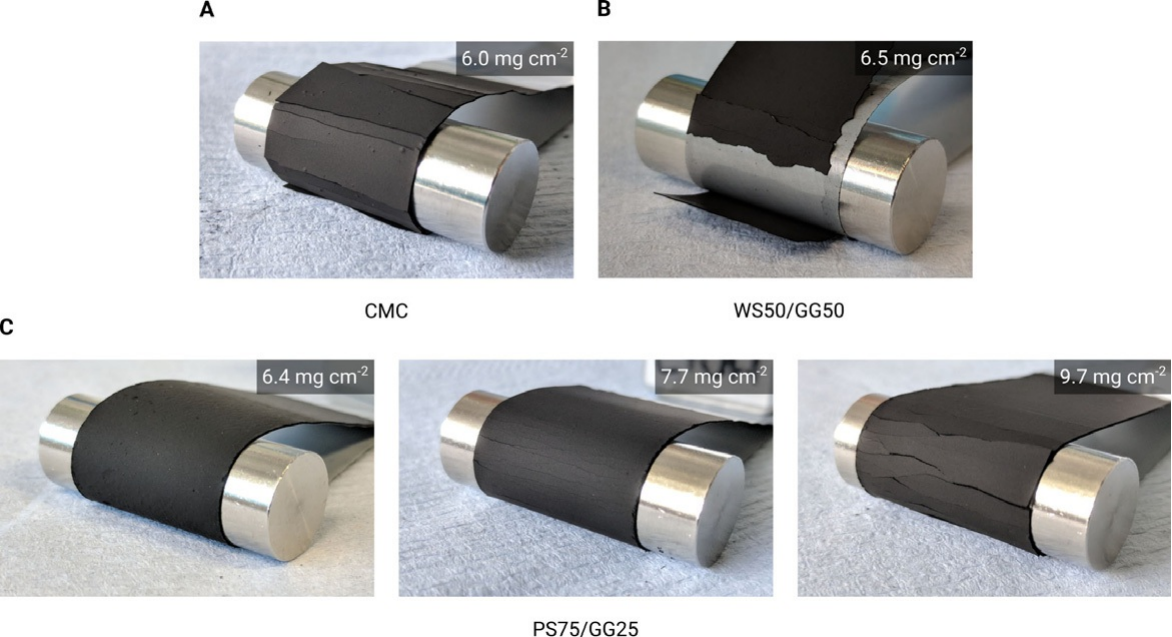
Photographs of bending tests. (A) Coating based on CMC. Severe cracks appeared already at medium to low mass loadings, showing that the flexibility is insufficient. (B) Coating based on WS50/GG50. The coating was not flexible enough and also delaminated at medium mass loadings. (C) Coatings based on PS75/GG25 with increasing mass loading. The first small cracks only appeared at approximately 7 mg cm^−2^.

To test the flexibility of the high‐mass‐loading coatings based on the new binders, the electrodes were cut into approximately 2 cm wide strips and wrapped around 12 mm diameter steel pins. It should be noted that the flexibility test used here was quite conservative, considering that rolls in industrial coating lines are typically several cm in diameter. However, this allowed us to compare the relative bendability of the electrodes featuring different binders.^15^ For the sake of comparison with the state of the art, CMC‐based electrodes were also subjected to the same procedure. As noticeable in Figure 1 A, high‐mass‐loading CMC‐based electrodes show large cracks. In fact, small‐to‐medium‐size cracks are already noticeable at loadings of approximately 3.5–4.0 mg cm^−2^. However, Figure 1 B shows that the WS50/GG50 mixture also tends to crack and delaminate at approximately 6 mg cm^−2^, precluding its use as a binder. In contrast, PS75/GG25 only shows minor cracks even up to 7 mg cm^−2^ mass loading, demonstrating much better flexibility. This is a strong indication that PS75/GG25 represents a significant improvement over the state of the art, enabling the high mass loadings requested at the industrial scale. Thus, the following investigations were focused on the PS75/GG25 mixture. Further details about the coating thicknesses and mass loadings of this mixture can be found in Figure S2 in the Supporting Information.

As a next step, the adhesion strength for both PS75/GG25 and CMC electrodes was determined. For PS75/GG25‐based electrodes the value is 400 kPa, but the limiting factor is the inner electrode cohesion rather than adhesion to the current collector. For CMC, in contrast, the mechanical stability of the CMC‐based electrodes is 1100 kPa, limited by the adhesion of the layer to the current collector. Nonetheless, the coating stability of the PS75/GG25 electrodes is deemed sufficient.

### Micro‐morphology

**Figure 2:**
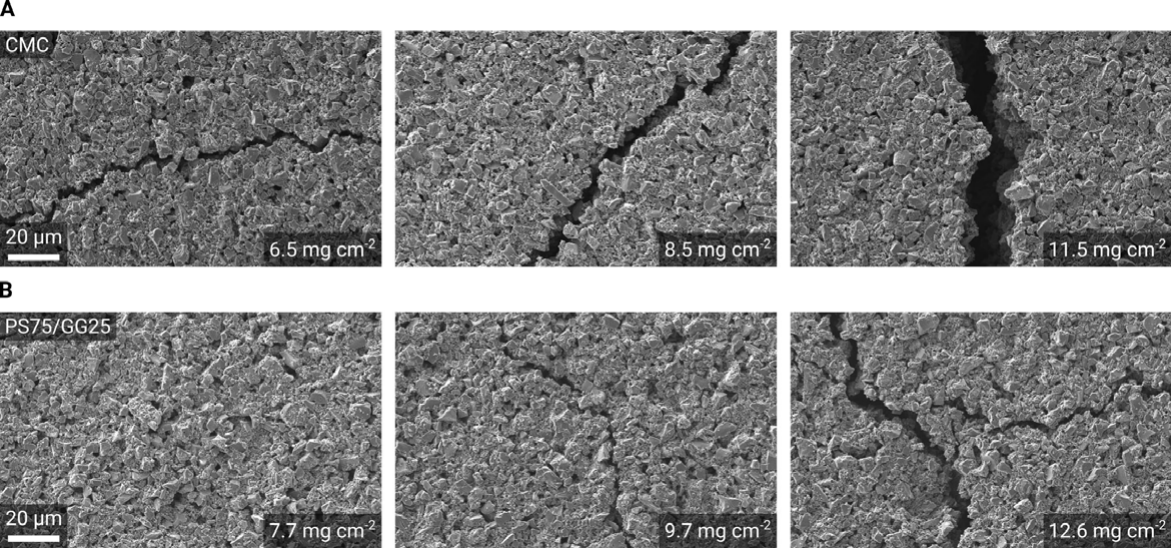
SEM images of coatings with different binders and mass loadings. (A) Cracks of increasingly larger size in CMC‐based coatings with increasing mass loadings. (B) PS75/GG25‐based coatings exhibited no cracks even at relatively high mass loadings and smaller cracks at very high mass loadings.

The morphology of the coated electrodes was investigated by SEM. These electrodes were not subjected to any mechanical stress but simply taken after drying. Figure 2 shows a series of SEM images comparing CMC‐ (Figure 2 A) and PS75/GG25‐based electrodes (Figure 2 B) with increasing mass loadings. It is clear that the number and size of cracks in the CMC‐based electrodes are larger than in those employing the PS75/GG25 binder, especially at high mass loadings. Indeed, the first cracks in PS75/GG25‐based electrodes only appear at very high mass loadings (9.7 mg cm^−2^).

It is well known that CMC tends to contract strongly during drying. This is clearly the reason for the greater extent of microscopic cracks compared with PS75/GG25.^3^ Microscopic cracking may promote accelerated disintegration of the electrode because parts of the active material layer can lose contact with the bulk of the electrode and the current collector. In fact, CMC‐based layers at higher mass loadings (≈7 mg cm^−2^) contract so strongly that macroscopic cracks appear when the layers are not allowed to relax and roll up under the tension. In the absence of constraints, as during the drying in industrial processes in which the foil is only supported by a hot air stream, the foil would strongly deform. However, this limitation does not seem to occur when using the mixture of PS and GG as binder.

Another important aspect in the electrode morphology is the microscopic homogeneity. A well‐dispersed conductive additive is essential to achieve low electrode resistance.^7^ If the interaction of the binder with either the active material or the conductive additive is too strong there is a risk of agglomeration. The surface of PS75/GG25‐based electrodes at high magnification shows well‐dispersed electrode components (see Figure S3 in the Supporting Information). These electrodes also show a rather good homogeneity along their thickness as demonstrated in Figure S4 in the Supporting Information, depicting the cross‐section of the electrode made by focused ion beam (FIB) milling.

### Thermal stability

**Figure 3:**
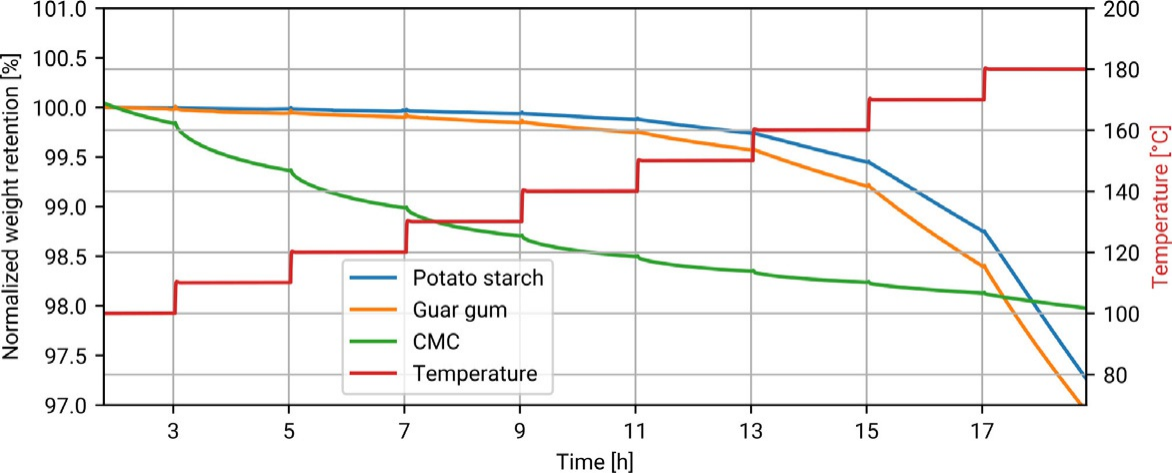
TGA of binder components. After initial water loss at 100 °C, PS and GG only showed insignificant mass loss at increased temperatures and for extended heating durations in air.

High temperatures are necessary for cost‐effective electrode production in short and fast drying lines, ensuring high productivity.^1^ However, if the binder degrades during the drying process, the integrity of the coating is compromised. Therefore, the thermal stability of the binder components is rather important and needs to be evaluated. For this purpose, thermogravimetric analysis (TGA) experiments were performed on the GG and PS separately, as well as CMC as a control. Because the binders adsorb significant amounts of water at room temperature,^16^ which might hide the weight loss associated with their decomposition, all samples were first subjected to a 3 h heating step at 100 °C to ensure the complete removal of water. To reliably determine the thermal stability, isothermal steps were used during TGA rather than a constant heating rate. The temperature was increased stepwise and held at each value for 2 h. As can be seen in Figure 3 A, even at 180 °C the mass loss for both PS and GG is only 0.7 % h^−1^. In an industrial production line, the electrodes would typically pass the drying stage within a few minutes.^15^ It follows that PS and GG are sufficiently stable in air at high temperatures to enable electrode production. Actually, these binders release most of the water already in the isothermal step at 100 °C, whereas CMC does not, meaning that they are much easier to dry than CMC.

### Electrochemical stability

With the production‐related hurdles for the PS75/GG25‐based electrodes having been passed, the electrochemical properties were investigated. First, the electrochemical inertness of the binder mixture itself at the relevant potentials employed in EDLCs had to be demonstrated. In fact, the binder(s) must not limit the usable operating voltage of the device owing to side reactions. Furthermore, side reactions might compromise the binding ability of the binder(s) and result in pulverization or delamination of the electrode, which in turn would cause capacity loss.^17^, ^18^

Two common state‐of‐the‐art electrolytes, tetraethylammonium tetrafluoroborate (TEA BF_4_) in acetonitrile (MeCN) or propylene carbonate (PC), were selected along with three novel ones developed by Balducci and co‐workers.^19^, ^20^, ^21^ These latter electrolytes were developed to improve conductivities and electrochemical stability windows beyond the state of the art, together with reduced toxicity, cost, and flammability. For example, *N*‐methyl‐*N*‐butylpyrrolidinium tetrafluoroborate (Pyr_14_ BF_4_) and 3‐cyanopropionic acid methyl ester (CPAME) combined with conventional salts and solvents are promising new electrolyte components.

The intrinsic stability of the binder was evaluated by cyclic voltammetry. The binder was first coated on the current collectors (mass loading ≈0.15 mg cm^−2^, see Figure S5 A in the Supporting Information) without any carbonaceous material to assess the faradaic currents originating from the binder decomposition, which would be hidden by the large capacitive current from a full carbon electrode.^22^, ^23^ The “binder” electrodes were used as working electrodes in three‐electrode cells by using a leakless reference Ag/AgCl 3.5 m KCl electrode for accurate determination of the onset potential of significant faradaic currents.^24^ Separate cells were assembled for cathodic and anodic sweeps. The sweeps started at −0.250 V versus Ag/AgCl 3.5 m KCl, which roughly corresponds to the initial potential of carbon,^25^ to ensure equal scope of probing of the electrochemical window in both the cathodic and anodic directions. Because a typical EDLC will go to the extreme potentials in its operating life many times, it is not reasonable to conclude limits from the first cycle current onset, in which only minor reactions associated with transient phenomena are typically observed. Hence, the sweeps were repeated ten times to show the development of reactivity at the edges of the potential window.

**Figure 4:**
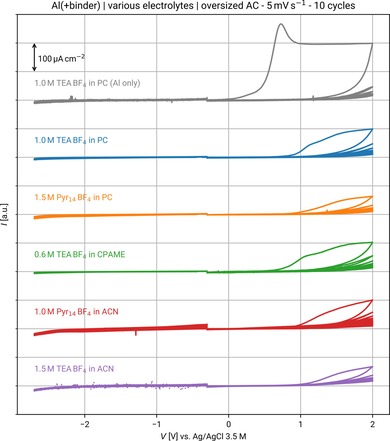
CV of the binder in several electrolytes. Cathodic and anodic CV sweeps at 5 mV s^−^1 of pristine and binder‐covered aluminum electrodes in the various electrolytes.

The top curve in Figure 4 shows the current response for a completely blank Al current collector. In this exemplary measurement, the anodic sweep exhibits an irreversible oxidation peak, resulting from Al dissolution, which decreases upon cycling as the result of passivation phenomena. This anodic dissolution peak is also found in the anodic sweeps of binder‐coated Al electrodes; however, the onset occurs at much higher potentials. Moreover, the anodic current recorded during the following cycles is lower, indicating a passivation occurs even with the coated binder covering the Al surface. Finally, the binder does not react to any appreciable extent with any of the employed electrolytes. Even at the highest probed potentials, the current response decays to a few tens of μA cm^−2^ after ten cycles, whereas the binder shows no sign of decomposition or delamination (see Figure S5 A–C in the Supporting Information).

These results demonstrate that the PS75/GG25 binder does not limit the electrochemical stability of the electrodes. Indeed, by using conventional binders and full carbon electrodes, Balducci and co‐workers found smaller potential windows, and smaller values are also reported for TEA BF_4_ in PC and MeCN in the literature.^19^, ^21^, ^26^

### Electrochemical impedance spectroscopy

**Figure 5:**
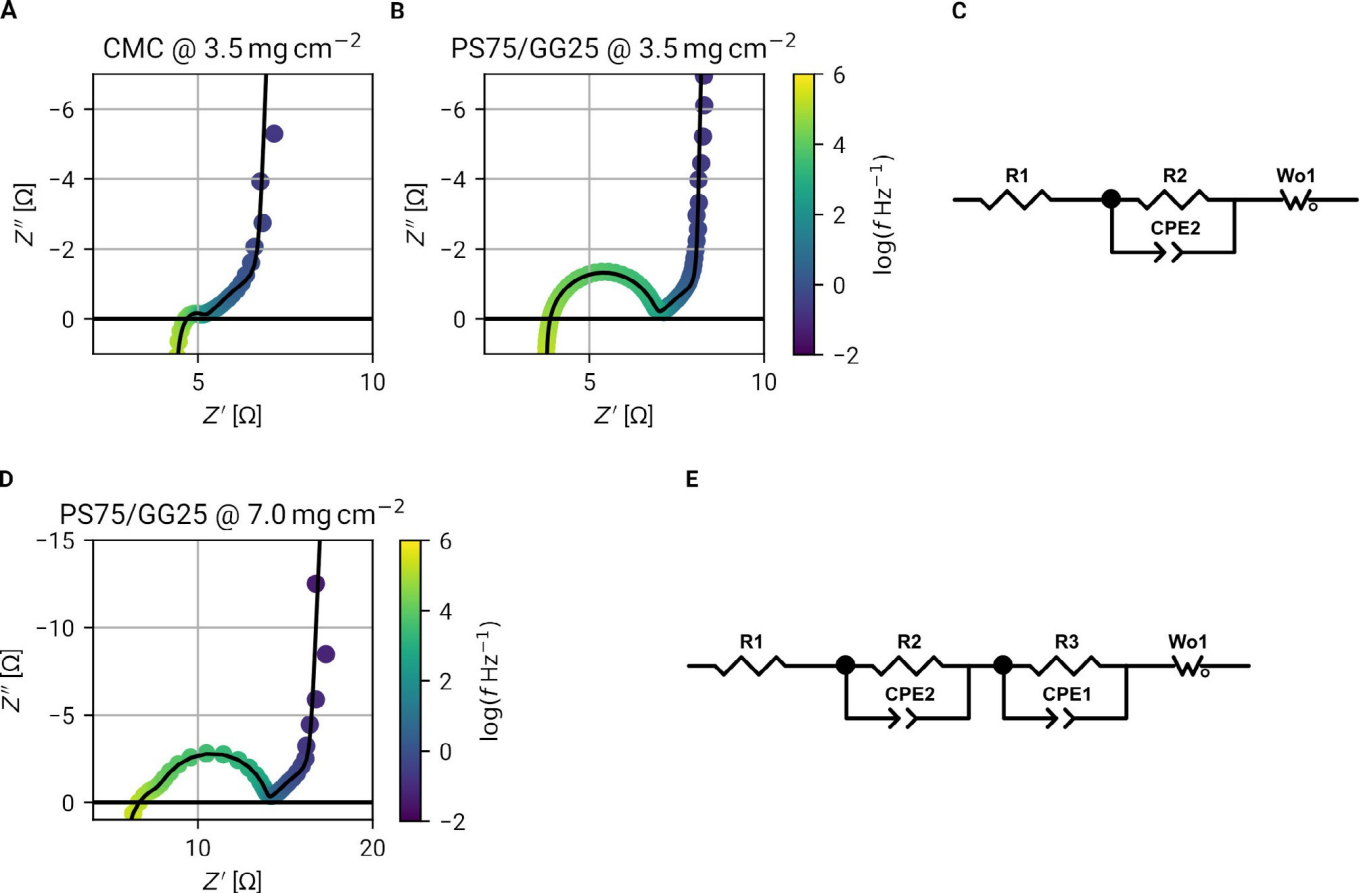
EIS of symmetric cells with electrodes based on CMC and PS75/GG25 in 1 m TEA BF_4_ in PC, *U*_DC_=0 V, *U*_AC_=5 mV. (A) Spectrum of CMC‐based electrodes with standard loading. (B) Spectrum of PS75/GG25‐based electrodes with standard loading. (C) Equivalent circuit model used to fit spectra in (A) and (B). The semicircles are related to interfacial resistance processes modeled by the RQ element (R2, CPE2). They are attributed to contact resistance between the carbon particles and the current collector. PS75/GG25 exhibits higher R2 values. (D) Spectrum of PS75/GG25‐based electrodes with high loading. (E) Equivalent circuit model used to fit spectrum in (D).

To characterize PS75/GG25‐based AC electrodes, in particular, the influence of the binder on the electrochemical behavior, electrochemical impedance spectroscopy (EIS) measurements were performed on symmetrical cells. Different cells were investigated employing CMC‐ and PS75/GG25‐based electrodes with 3.5 mg cm^−2^ active material loading (designated as “standard loading”) and 7.0 mg cm^−2^ (designated as “high loading”; these electrodes were achieved only with PS75/GG25). The corresponding EIS results are shown in Figure 5. Although the standard loading electrodes employing the two binders show different total impedance (Figure 5 A, B), their impedance spectra could be described with the same equivalent circuit (see Figure 5 C). Besides the expected electrolyte resistance at high frequencies, modeled by R1, and the diffusion‐limited/capacitive response described by the open Warburg element Wo1 (at low frequencies), the semicircle at medium frequencies is indicative of charge‐transfer processes at an interface. This was modeled by a parallel RQ element comprising a resistance (R2) and constant phase element (CPE2). In the case of CMC, the value for R2 is approximately 1 Ω, whereas R2 equals 3 Ω for PS75/GG25‐based electrodes. Interestingly, in high‐loading‐type cells (see Figure 5 D), two distinguishable semicircles could be resolved in the same frequency range. Therefore, two separate RQ elements were employed to fit this spectrum (see model EC in Figure 5 E). Here, the values are 2 Ω for R2 and 5 Ω for R3. These semicircles are attributed to the transfer of electrons between the active material particles and the electrode coating and the current collector.^27^, ^28^ In the case of CMC, the interfacial resistances are relatively low, but PS75/GG25 exhibits slightly higher resistance contributions from both sources. Because the interfacial resistance at the current collector should be independent from the mass loading, it is reasonable to attribute the second RQ element (R3|CPE3, resolved only for high loading at lower frequencies) to the particle–particle interfaces.

In summary, the EIS results indicate a slight drawback of PS75/GG25 versus CMC in terms of electrode resistance, well matching the FIB cross‐section observation (see Figure S4 in the Supporting Information), indicating that the electrode layer/current collector contact still needs to be improved.

### Long‐term cycling

Although the three‐electrode measurements excluded evident detrimental reactions of the binder with any of the tested electrolytes, it is also important to assess the performance of full electrodes in two‐electrode cells over longer timescales. This helps to rule out other possible issues such as swelling of the binder or slow degradation.^5^ In addition, as the EIS measurements illustrated, the binder has a major impact on the electrode resistance, which could be detrimental for cycling or rate performance.^7^

Very often in the literature, EDLCs are tested with cycling protocols that charge and discharge the cell repeatedly, usually more than 10 000 times, but without any constant voltage step at full charge. Even with slow charging rates, this results in the EDLC spending only a small fraction of time at high voltages.^29^ Consequently, the long‐term stability is often overestimated. In industry, however, testing protocols typically involve long steps at high voltage. The protocol used here was directly adapted from a European manufacturer of EDLCs and based on IEC standard 6239/1.^30^ Further details can be found in the Supporting Information.

**Figure 6:**
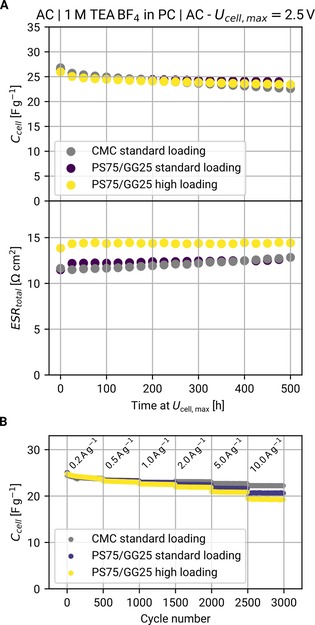
Cycling performance of symmetric cells. (A) Capacitance retention and ESR increase over the course of voltage‐hold experiments. PS75/GG25‐based electrodes showed identical capacitance compared to CMC‐based ones at standard mass loading (3.5 mg cm^−2^), and electrodes with high mass loadings (7.0 mg cm^−2^) only showed a minor increase in ESR but identical capacitance retention. (B) Rate performance of electrodes with different binders and mass loadings.

Here, symmetric coin cells with 1 m TEA BF_4_ in PC were assembled with electrodes based on CMC or PS75/GG25. Once more, cells employing different binders (CMC and PS75/GG25) and different electrode loadings (3.5 and 7.0 mg cm^−2^, “standard” and “high loading”, respectively) were tested. The results of the voltage hold test can be seen in Figure 6 A. It is evident that the PS75/GG25 enables a performance equivalent to CMC in terms of capacitance retention. This is even true for the high‐loading electrodes, which only show a slightly increased equivalent series resistance (ESR). Therefore, such high‐loading electrodes can significantly increase the active material/current collector ratio without penalizing the performance. If one includes the current collector mass into the calculation (5.18 mg cm^−2^ at the thickness of 20 μm employed here), this represents a specific energy gain of approximately +45 % at the electrode level.

The cycling performance of the electrodes was also tested by using the same type of coin cells but employing fast constant current charge/discharge cycles (i.e., without voltage hold steps). The results, illustrated in Figure 5 B, show that the rate performance of PS75/GG25‐based electrodes is mostly equivalent to CMC‐based ones. The capacitance for all cells is only moderately affected, and only at very high currents. Overall, the cycling data strongly suggests that PS75/GG25 is basically identical to CMC but allows doubling the mass loading.

## Conclusions

Three natural binder materials for electrochemical double‐layer capacitors (EDLCs), guar gum (GG), potato starch (PS), and wheat starch were investigated and compared against the widely used aqueous binder carboxymethyl cellulose (CMC). The 3:1 mixture of PS and GG resulted in the most favorable rheology for coating, enabling high solid contents and flexible high‐mass‐loading electrodes. PS75/GG25 could survive bending tests at up to 7 mg cm^−2^, twice as much as CMC. SEM microscopy showed good dispersion of the active material and conductive additive, as well as reduced crack formation during drying. Both PS and GG were shown to be stable at the typical drying temperatures in air, enabling fast drying. Binder‐covered current collectors were used as working electrodes in cyclic voltammetry experiments to determine their electrochemical stability in both state‐of‐the‐art and novel electrolytes. They showed excellent stability windows that were larger than that of activated carbon electrodes, proving that they would not limit the devices′ operating voltage. Electrochemical impedance spectroscopy was employed to further characterize the PS75/GG25 electrodes, and a slightly increased contact resistance compared with CMC was observed. Symmetric full cells were subjected to long‐term voltage‐hold experiments. PS75/GG25‐ and CMC‐based electrodes showed identical and excellent capacitance retention with only minor equivalent series resistance increase. Doubling the mass loading for PS75/GG25 had only a small impact on performance but represents a large increase in specific capacitance on the full electrode level, owing to the lower share of current collector weight. The rate capability for PS75/GG25 was also very similar to CMC‐based electrodes. PS/GG‐based binders thus are a promising alternative to CMC.

## Experimental Section

### Electrode preparation and characterization

Potato starch (PS) was purchased from Sigma–Aldrich. Wheat starch (WS) and guar gum (GG) were kindly supplied by Kröner Stärke GmbH and Lamberti S.p.a, respectively. CMC was purchased from Dow Wolff Cellulosics (CMC Walocell 2000 PA). Activated carbon (AC, YP50 from Kuraray) and etched aluminum foil (20 μm, 5.18 mg cm^−2^) were kindly supplied by Skeleton Technologies GmbH. Carbon black (CB) was purchased from Imerys Graphite & Carbon (C‐ENERGY Super C45). All slurries were prepared with weight ratios of AC/CB/binder of 90:5:5. The solid content was adjusted as needed to achieve homogeneous coatings. The binder was stirred in ultrapure water (milli‐Q) until fully dispersed and, in the case of PS, heated for 30 min at 70 °C. CB was added next, and the slurry was stirred for at least 1 h. Water lost during stirring was added again. AC was mixed in by manual mixing and kneaded until the slurry was well homogenized.

The slurries were coated onto Al foils with an adjustable doctor blade at 50 mm s^−1^. Coatings were allowed to dry at room temperature for approximately 30 min and then dried overnight at 80 °C. Bending tests were done with 12 mm diameter steel pins and 2 cm wide strips cut from dried electrodes. Mass loadings were determined by cutting 12 mm disks from the strips and weighing them.

Counter electrodes were made with weight ratios of AC/CB/polytetrafluoroethylene (PTFE) of 80:10:10. Using the 60 wt % PTFE solution in water (Sigma–Aldrich), CB and AC were dispersed by stirring until the water evaporated and a dough‐like slurry was formed. The slurry was rolled out to obtain a thick layer (about 0.5 mm), which was cut into 12 mm diameter disks.

TGA was done in synthetic air (20 % O_2_, 80 % N_2_) by using the Discovery TGA by TA Instruments. SEM images were taken with a Zeiss LEO 1550 microscope at 3 kV, using combined backscattered electron and secondary electron imaging.

### Electrochemical characterization

Tetraethylammonium tetrafluoroborate (TEA BF_4_), acetonitrile (MeCN), and propylene carbonate (PC) were purchased from Sigma–Aldrich. *N*‐Methyl‐*N*‐butylpyrrolidinium tetrafluoroborate (Pyr_14_ BF_4_) and 3‐cyanopropionic acid methyl ester (CPAME) based electrolytes were kindly supplied by the group of Prof. Andrea Balducci (Friedrich‐Schiller‐University Jena). Electrodes were cut into 12 mm disks and dried at 110 °C under vacuum overnight and transferred to an argon‐filled glovebox (LabMaster, Mbraun GmbH) with <0.1 ppm O_2_ and <0.1 ppm H_2_O for all cell assembly. Swagelok‐type three‐electrode cells were used for the CV experiments. AC‐overloaded (in terms of capacitance) counter electrodes were employed. As reference electrodes, Ag/AgCl 3.5 m KCl leakless minielectrodes (edaq) were used. Approximately 300 μL of electrolyte was impregnated into glass fiber disks (GF/D, thickness: 670 μm, diameter: 13 mm diameter; Whatman). A potentiostat/galvanostat (VMP3, Biologic Science Instruments) was employed to record the CVs. Two‐electrode EDLCs were assembled in coin cells with symmetric mass loadings of either 3.5 mg cm^−2^ (standard loading) or 7.0 mg cm^−2^ (high loading), by using 150 μL of electrolyte impregnated in the glass fiber separator. These were cycled in a Maccor Battery Tester 4300. EIS experiments were performed in symmetric two‐electrode Swagelok cells with an Impedance/Gain‐Phase Analyzer 1260 (Solartron Analytical). All electrochemical tests were performed in climatic chambers at *T*=(20±2) °C (KB115, Binder GmbH).

## Conflict of interest

*The authors declare no conflict of interest*.

## Supporting information

As a service to our authors and readers, this journal provides supporting information supplied by the authors. Such materials are peer reviewed and may be re‐organized for online delivery, but are not copy‐edited or typeset. Technical support issues arising from supporting information (other than missing files) should be addressed to the authors.

Supplementary

## References

[1] C. Schütter , S. Pohlmann , A. Balducci , Adv. Energy Mater. 2019, 9, 1900334.

[2] N. Böckenfeld , S. S. Jeong , M. Winter , S. Passerini , A. Balducci , J. Power Sources 2013, 221, 14–20.

[3] A. Varzi , S. Passerini , J. Power Sources 2015, 300, 216–222.

[4] H. Chen , M. Ling , L. Hencz , H. Y. Ling , G. Li , Z. Lin , G. Liu , S. Zhang , Chem. Rev. 2018, 118, 8936–8982. 30133259 10.1021/acs.chemrev.8b00241

[5] D. Bresser , D. Buchholz , A. Moretti , A. Varzi , S. Passerini , Energy Environ. Sci. 2018, 11, 3096–3127.

[6] M. Yamagata , S. Ikebe , K. Soeda , M. Ishikawa , RSC Adv. 2013, 3, 1037–1040.

[7] H. Y. Tran , M. Wohlfahrt-Mehrens , S. Dsoke , J. Power Sources 2017, 342, 301–312.

[8] N. Singh , J. Singh , L. Kaur , N. S. Sodhi , B. S. Gill , Food Chem. 2003, 81, 219–231.

[9] V. Vamadevan , E. Bertoft , Starch/Staerke 2015, 67, 55–68.

[10] R. J. Chudzikowski , J. Soc. Cosmet. Chem. 1971, 22, 43–60.

[11] V. Sudhakar , R. S. Singhal , P. R. Kulkarni , Food Hydrocolloids 1996, 10, 329–334.

[12] S. W. Cui , M. A. N. Eskin , Y. Wu , S. Ding , Adv. Colloid Interface Sci. 2006, 128–130, 249–256. 10.1016/j.cis.2006.11.012 17196539

[13] S. Nandi , P. Guha , Carbohydr. Polym. 2018, 200, 498–507. 30177191 10.1016/j.carbpol.2018.08.028

[14] L. Zhang , Z. Liu , G. Cui , L. Chen , Prog. Polym. Sci. 2015, 43, 136–164.

[15] A. Kwade , W. Haselrieder , R. Leithoff , A. Modlinger , F. Dietrich , K. Droeder , Nat. Energy 2018, 3, 290–300.

[16] X. Liu , Y. Wang , L. Yu , Z. Tong , L. Chen , H. Liu , X. Li , Starch/Staerke 2013, 65, 48–60.

[17] P. W. Ruch , D. Cericola , A. Foelske , R. Kötz , A. Wokaun , Electrochim. Acta 2010, 55, 2352–2357.

[18] D. Cericola , P. W. Ruch , A. Foelske-Schmitz , D. Weingarth , R. Kötz , Int. J. Electrochem. Sci. 2011, 6, 988–996.

[19] M. Arnaiz , E. Goikolea , T. Rojo , L. Wittscher , A. Balducci , J. Ajuria , J. Power Sources 2019, 434, 226757.

[20] P. Gerlach , R. Burges , A. Lex-Balducci , U. S. Schubert , A. Balducci , Electrochim. Acta 2019, 306, 610–616.

[21] S. Pohlmann , C. Ramirez-Castro , A. Balducci , J. Electrochem. Soc. 2015, 162, A5020–A5030.

[22] D. Weingarth , H. Noh , A. Foelske-Schmitz , A. Wokaun , R. Kötz , Electrochim. Acta 2013, 103, 119–124.

[23] M. P. S. Mousavi , A. J. Dittmer , B. E. Wilson , J. Hu , A. Stein , P. Bühlmann , J. Electrochem. Soc. 2015, 162, A2250–A2258.

[24] *Leakless Miniature Ag/AgCl Reference Electrode (Model ET072)*, https://www.edaq.com/product_sheets/transducers/ET072_Leakless_Miniature_Ag-AgCl_Reference_Electrode.pdf, **2009**.

[25] P. W. Ruch , D. Cericola , M. Hahn , R. Kötz , A. Wokaun , J. Electroanal. Chem. 2009, 636, 128–131.

[26] Supercapacitors: Materials, Systems and Applications (Ed.: M. Lu), Wiley-VCH, Weinheim, 2013.

[27] Y. R. Nian , H. Teng , J. Electroanal. Chem. 2003, 540, 119–127.

[28] S. Dsoke , X. Tian , C. Täubert , S. Schlüter , M. Wohlfahrt-Mehrens , J. Power Sources 2013, 238, 422–429.

[29] D. Weingarth , A. Foelske-Schmitz , R. Kötz , J. Power Sources 2013, 225, 84–88.

[30] *Instructions for Testing of Skeleton Technologies’ Ultracapacitors*, https://www.skeletontech.com/hubfs/171101_TestingInstructionsForUltracapacitors-1.pdf?hsLang=en, **2017**.

