# Supplementary material for: Natural Polymers as Green Binders for High‐Loading Supercapacitor Electrodes
Source: ChemSusChem. 2020 Jan 21;13(4):763–70. doi: 10.1002/cssc.201902863 (PMC7065209; doi:10.1002/cssc.201902863)
Supplement: Supplementary file 1 — Supplementary [file CSSC-13-763-s001.pdf]

CHEMISTRY & SUSTAINABILITY
CHEMSUSCHEM
ENERGY & MATERIALS

Supporting Information

Natural Polymers as Green Binders for High-Loading Supercapacitor Electrodes

Peter Ruschhaupt,[a, b] Alberto Varzi,*[a, b] and Stefano Passerini*[a, b]

cssc_201902863_sm_miscellaneous_information.pdf

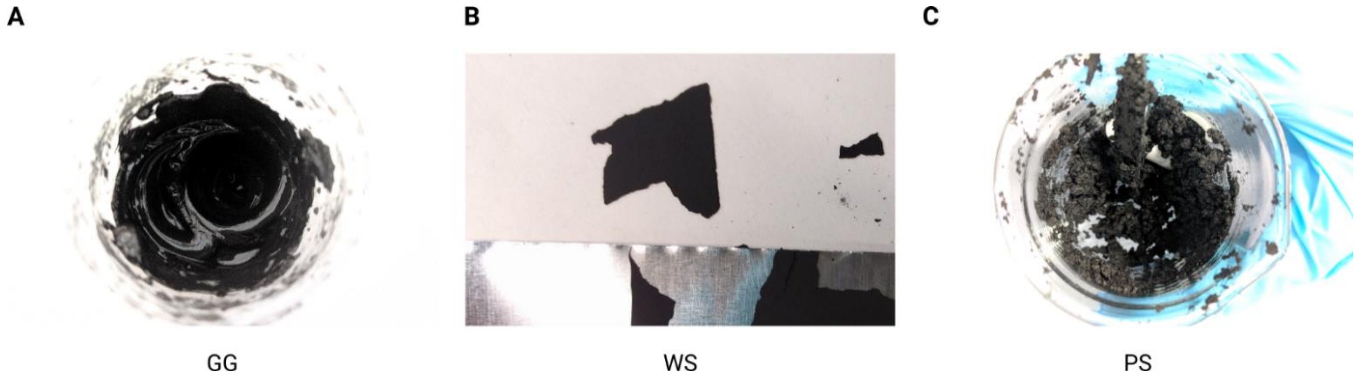

Fig. S1 ***Exclusion reasons of binder candidates*** *(A) Over 20 wt% solid content GG is not fluid enough to be coated. (B) WS coatings are brittle and have low adhesion even at low mass loadings (~1 mg cm*$^{-2}$*) (C) WS and PS (shown here) cannot be properly mixed and do not yield a liquid slurry over 25 wt%.*

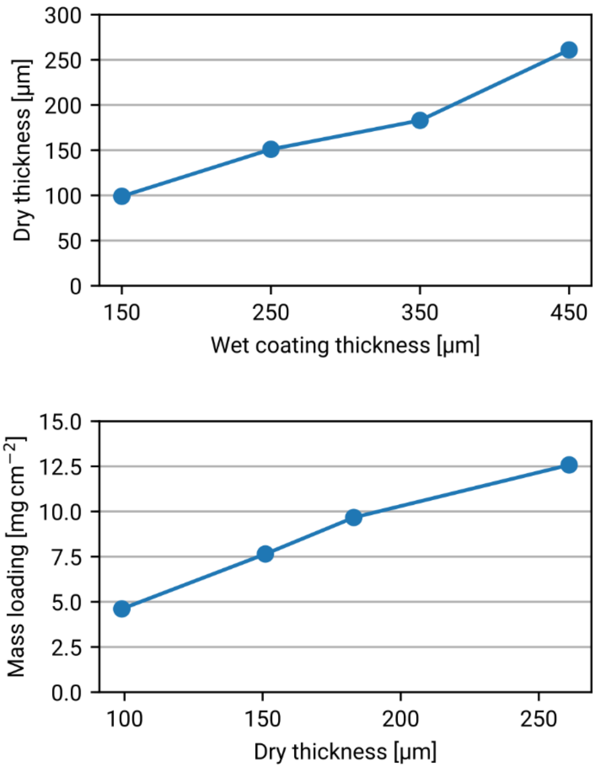

Fig. S2 ***Coating thickness data*** *Relationship of wet and dry thickness and mass loading using PS75/GG25 are mostly linear. Density of the coatings is 0.5 g cm*$^{-3}$*.*

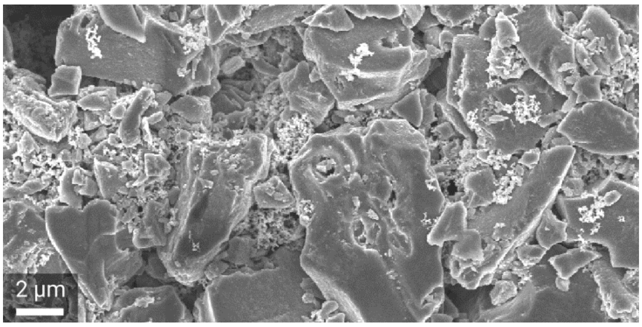

Fig. S3 ***Closeup of PS75/GG25 electrode*** *The conductive additive is well dispersed and shows no sign of agglomeration.*

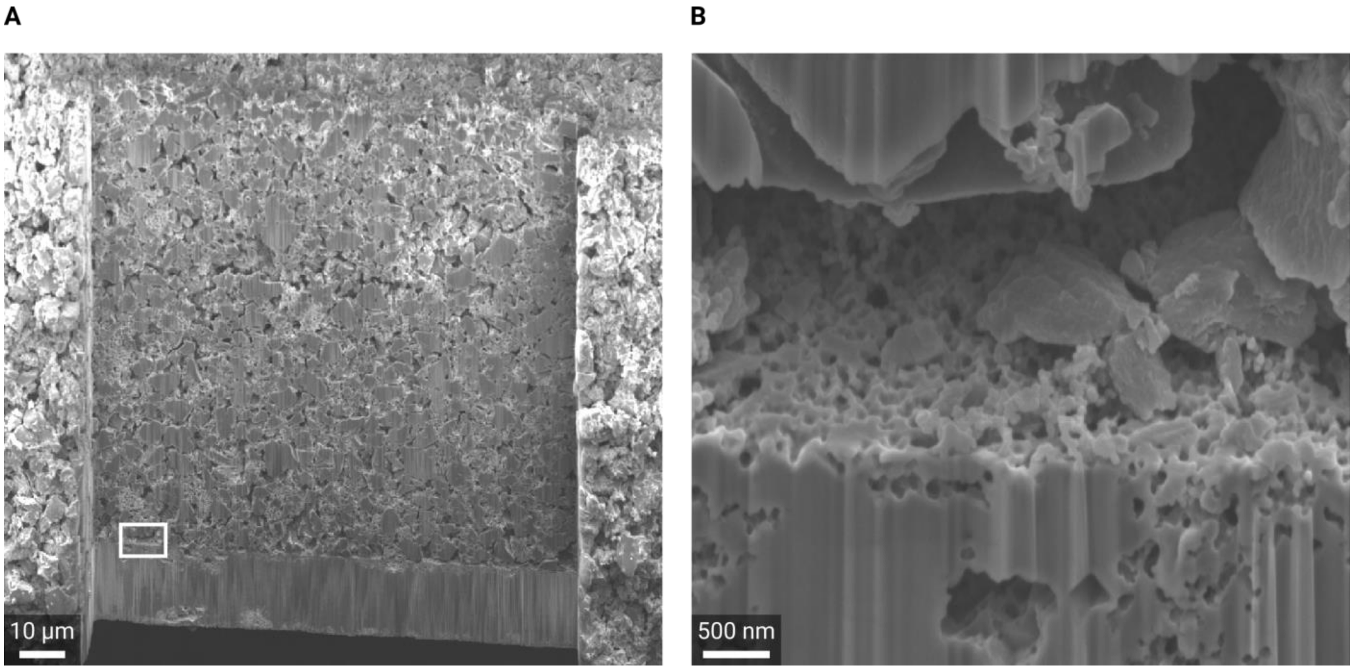

Fig. S4 ***Focused ion beam cross section*** *(A) Morphology along the entire thickness of the coating is homogenous. The white rectangle indicates the location of the magnified view. (B) Magnified view. Some space remains at the current collector coating interface that is likely to increase the interfacial resistance.*

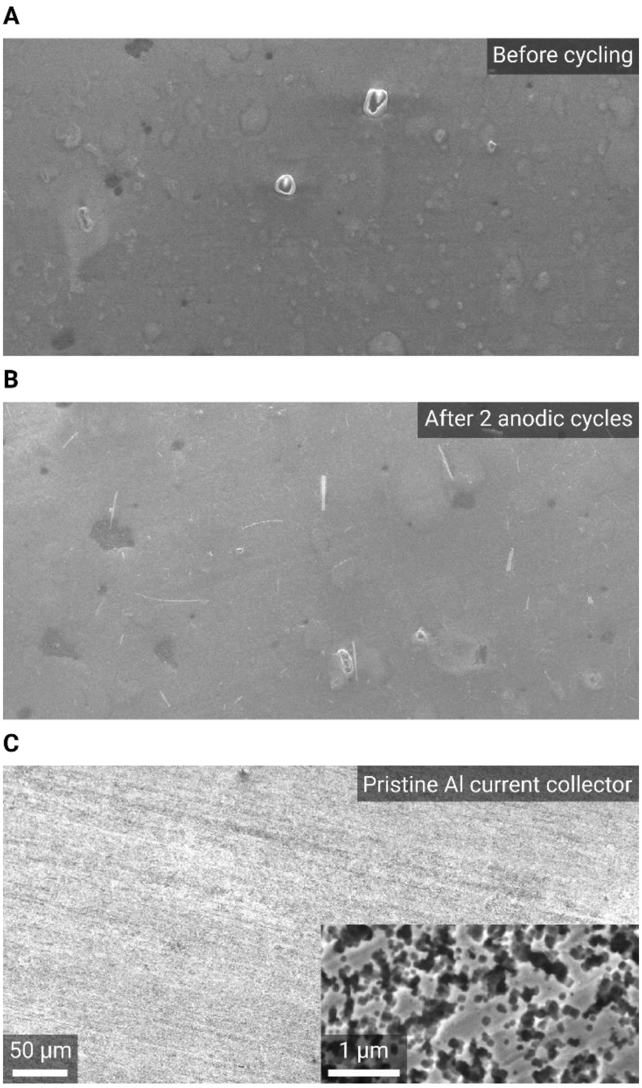

Fig. S5 ***Binder covered current collector used as WE for stability test.*** *(A) Current collector coated with 0.15 mg cm*$^{-2}$ *of PS75/GG25. The rough aluminum surface is not visible anymore. The visible particles are residual starch granules that did not dissolve. (B) Coated current collector after two anodic CV cycles to 2 V vs. Ag/AgCl 3.5 M KCl. No delamination or other changes can be observed, except for the adhesion of some residual glass fibers from the separator. (C) Pristine Al current collector with roughened surface for comparison to (A) and (B). Inset shows magnified view of surface structure.*

Cycling protocol

The EDLC is kept at its maximum operating voltage for 500 h. Every 25 h the capacitance and equivalent series resistance (ESR) are determined with five slow constant current charge-discharge steps (see Fig. S6 for details). In this way the capacitance is undistorted from any effects from high currents. In this work the specific capacitance is calculated by dividing by the total coating mass including active material, conductive additive, and binder. The EDLC was discharged to half of the operating voltage only, in order to probe the 75% of energy storage that is typically used in EDLCs. The cells tested here showed almost perfectly ideal linear behavior (see Fig. S6). To avoid any residual effects from the voltage hold step, the capacitance and ESR values are determined from the average of the last three cycles.

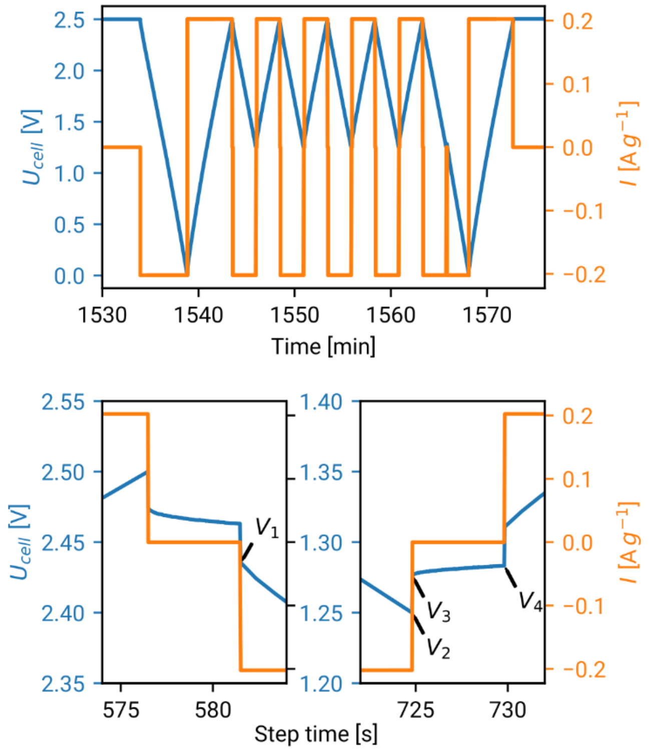

Fig. S6 ***Cycling performance test scheme*** *Devices are held at maximum voltage for 25 h and then charged and discharged five times to determine capacitance and equivalent series resistance. The black rectangles indicate the location of the lower enlarged graphs, showing the relevant voltages. From* V_1 *and* V_2 C *can be determined.* V_3 *and* V_4 *are used to determine resistance from the voltage rise after current stops.*

The capacitance is calculated via the following formula.

$$C = \frac{V_2 - V_1}{t_2 - t_1}$$

The ESR can be calculated from the voltage rise when stopping the current.

$$ESR_{nom} = \frac{V_3 - V_2}{I}$$

$$ESR_{tot} = \frac{V_4 - V_2}{I}$$

Here the first voltage rise represents fast electric relaxation effects while the slower rise over 5 s includes slower processes in the electrolyte and pores.
